# Different histopathologic profiles and outcomes between sun-exposed BCC and non-sun-exposed BCC

**DOI:** 10.1038/s41598-020-64391-9

**Published:** 2020-04-30

**Authors:** Chia-Lun Li, Yu-Ching Chen, Kuo-Chung Yang, Lee-Wei Chen

**Affiliations:** 1 Department of Surgery, Kaohsiung Veterans General Hospital, Kaohsiung, Taiwan; 2 School of Medicine, National Yang-Ming University, Taipei, Taiwan; 3 Department of Biological Sciences, National Sun Yat-Sen University, Kaohsiung, Taiwan; 4 Institute of Emergency and Critical Care Medicine, National Yang-Ming University, Taipei, Taiwan

**Keywords:** Skin cancer, Skin cancer

## Abstract

Asian population is a low-risk group for basal cell carcinoma (BCC) and there is little data available in this setting. Sun-exposed BCC (SEBCC) may possess a different pathogenic mechanism from non-sun-exposed BCC (NSEBCC). To compare the histopathological profiles and outcomes between SEBCC and NSEBCC, and to assess the risk factors for tumor recurrences. Retrospective cohort study on 372 patients with pathologically diagnosed BCC from January 1, 1990 to August 31, 2017. Data were derived from a single medical center in Taiwan. SEBCC presented with higher Clark level and more high-risk factors for recurrence than NSEBCC. Nodular, micronodular, infiltrating/mixed infiltrating, basosquamous, and adenoid types were predominant in SEBCC, as superficial type in NSEBCC. Risk factors for recurrence included infiltrating/mixed-infiltrating subtypes and synchronous basosquamous cell carcinoma. No recurrence events were observed in NSEBCC. Our study showed an acceptable recurrence rate (4.2%) of the whole population after excision even under a smaller surgical margin width than suggested by current guidelines. SEBCC had a higher recurrence rate with a significantly different tumor characteristic from NSEBCC and a greater tumor depth than NSEBCC. A wider surgical margin in SEBCC than NSEBCC is suggested.

## Introduction

Basal cell carcinoma (BCC) is the most common skin cancer and has an increasing incident worldwide^1^. Although the incidence is relatively low (2-4%) in Asian populations compared to Caucasian ones, studies have suggested a disproportionately high morbidity and mortality associated with the diagnosis of BCC in people with skin of color^2–4^. There is little data available about the characteristics and outcomes of BCC in Asian people. In Taiwan, non-melanoma skin cancer (NMSC) has often been ranked among the top ten most common types of cancer^5^.

Ultraviolet (UV) radiation has been viewed as one of the most important factors of BCC development^6–8^, as various epidemiological studies found the most common anatomical site of BCC to be head and neck. On the other hand, the pathogenesis of BCC on non-sun-exposed body area remains unclear. Studies have hypothesized a follicular stem cell or hair bulge origin of BCC^9,10^. Other factors including radiation, intermittent UV light exposure, chemical carcinogens, and infection with human papillomaviruses have also been reported^11–14^. To this date, most studies regarding the histopathologic features and outcomes of BCC were grouped by anatomical site rather than by areas that are prone to sun-exposure. Considering the potential differences in pathogenesis between sun-exposed BCC (SEBCC) and non-sun-exposed BCC (NSEBCC), this study aims to compare the histopathologic profiles and outcomes between SEBCC and NSEBCC and to investigate the risk factors for tumor recurrence.

## Results

Of all 372 patients, 338 (90.9%) had SEBCC while the other 34 (9.1%) had NSEBCC. The mean age (±standard deviation) of index lesion was 71.8 (±13.7) years and 70.5 (±12.1) years in the SEBCC group and the NSEBCC group, respectively. No significant differences were found regarding the length of follow-up in the two groups (4.93 vs 4.51 years). Patients in the NSEBCC group had a significantly higher association with chronic kidney disease (CKD) (17.6% vs 7.1%, p < 0.001) and rheumatoid arthritis (RA) (5.9% vs 0%, p = 0.031) than with those in the SEBCC group. Details of the characteristics and comorbidities of the population are described in Table 1.

**Table 1:** Study population characteristics (n = 372).

	Patient with SEBCC	Patient with NSEBCC	p-value
Patient number (%)	338 (90.9%)	34 (9.1%)	—
Men/ Women (n)	213/125	23/11	p = 0.593
Age at onset, mean ± SD (range)	71.8 ± 13.7 (11-99)	70.5 ± 12.1 (43-86)	p = 0.576*
Duration of follow up (year), mean ± SD (range)	4.51 ± 4.2 (0-26)	4.94 ± 4.3 (0-19)	p = 0.564*
Co-morbidities (%)			
DM	89 (26.3)	7 (20.6)	p = 0.466
HT	156 (46.2)	17 (50.0)	p = 0.668
CKD	24 (7.1)	6 (17.6)	p = 0.031
ESRD	11 (3.3)	0 (0)	p = 0.609
CVA	37 (10.9)	1 (2.9)	p = 0.231
CAD	29 (8.6)	2 (5.9)	p = 0.999
Arrhythmia	22 (6.5)	3 (8.8)	p = 0.489
VHD	5 (1.5)	0 (0)	p = 0.999
ILD	3 (0.9)	0 (0)	p = 0.999
Asthma	2 (0.6)	0 (0)	p = 0.999
COPD	4 (1.2)	0 (0)	p = 0.999
Dementia	11 (3.3)	2 (5.9)	p = 0.337
Heart failure	7 (2.1)	1 (2.9)	p = 0.539
RA	0 (0)	2 (5.9)	p = 0.008
Gout	8 (2.4)	3 (8.8)	p = 0.069
Hyperthyroidism	1 (0.3)	1 (2.9)	p = 0.175
HBV infection	7 (2.1)	1 (2.9)	p = 0.539
HCV infection	9 (2.7)	1 (2.9)	p = 0.999
Hepatitis	3 (0.9)	1 (2.9)	p = 0.320
Liver cirrhosis	5 (1.5)	0 (0)	p = 0.999
Anemia	6 (1.8)	2 (5.9)	p = 0.160
Tuberculosis	1 (0.3)	1 (2.9)	p = 0.175
GI disease	36 (10.7)	0 (0)	p = 0.060
Arsenism	6 (1.8)	2 (5.9)	p = 0.160
Immunocompromised	121 (35.8)	17 (50)	p = 0.135

DM: diabetes mellitus; HT: hypertension; CKD: chronic kidney disease; ESRD: end-stage renal disease; CVA: cerebral vascular accident; CAD: coronary artery disease; VHD: valvular heart disease; ILD: interstitial lung disease; COPD: chronic obstructive pulmonary disease; RA: rheumatoid arthritis; HBV: hepatitis B virus; HCV: hepatitis C virus; GI: gastrointestinal; N: number; SD: standard deviation. Using two sample t-test (*), otherwise chi-square test or Fisher’s exact test. p-value <0.05: two-tailed statistical significance.

From the pathology reports, we identified 403 primary BCC lesions among 372 patients (Table 2). The most commonly diagnosed sites were on the head and neck (88.1%), with the most common subsites being nose or surroundings of the nose (29.5%), followed by cheeks and zygomatic area (13.9%), then eyebrows or area surrounding the eye (12.4%). Of the remaining, 6.0% of the lesions were from the trunk, 4.5% from extremities, and 1.5% from the other sites.

**Table 2:** Tumor site of all BCC lesions.

Tumor site (N = 403 BCCs)	No. of BCCs (%)
Head and neck	355 (88.1)
Nose or surroundings of nose	119 (29.5)
Nasolabial fold	19 (4.7)
Cheeks and zygomatic area	56 (13.9)
Lips and supralabial area	13 (3.2)
Eyebrows or area surrounding the eye	50 (12.4)
Forehead and glabella	12 (3.0)
Temporal areas	7 (1.7)
Ears and periauricular areas	35 (8.7)
Chin	6 (1.5)
Scalp	32 (7.9)
Neck	6 (1.5)
Trunk	24 (6.0)
Back and shoulder	10 (2.5)
Axillary	2 (0.5)
Thorax	6 (1.5)
Abdomen	6 (1.5)
Extremities	18 (4.5)
Upper arm	2 (0.5)
Forearm	3 (0.7)
Wrist	2 (0.5)
Thigh	7 (1.7)
Lower leg	3 (1.7)
Popliteal	1 (0.2)
Other, pelvic, anogenital area, or buttocks	6 (1.5)

### Risk factors for multiple BCC

22 (5.9%) patients were recorded with multiple BCCs, including 18 from the SEBCC group and 4 from the NSEBCC group. The associations between whether the lesions being sun-exposed or not, patient demographics, comorbidities, and the development of multiple BCCs using single and multivariate logistic regression analyses are shown in Table 3. Patients with dementia had a significantly higher association with the development of multiple BCCs (aOR=5.84, CI = 1.34-25.58, p = 0.019). Although patients with NSEBCC tended to have a higher association with developing multiple lesions than those with SEBCC, no significant differences were found. No significant associations regarding age, sex, diabetes mellitus, hypertension, chronic kidney disease, gout, and Arsenism were found to affect the development of multiple BCCs.

**Table 3:** The crude and adjusted odds ratio of area of BCC, patient demographics, or comorbidities associated with the development of multiple BCCs by logistic regression analyses.

		Multiple, n (%)	Singular, n (%)	COR (95% CI)	p-value	AOR (95% CI)	p-value
Lesion of sun-exposed area		18 (81.8)	320 (91.4)	0.42 (0.13-1.33)	0.140	0.55 (0.16-1.90)	0.342
Age	<65.0	3 (13.6)	91 (26.0)	1 [Reference]			
	65.0–74.99	7 (31.8)	85 (24.3)	2.45 (0.63–9.97)	0.195	2.43 (0.58–10.25)	0.228
	>=75.0	12 (54.5)	174 (49.7)	2.09 (0.58–7.60)	0.262	2.03 (0.51–8.13)	0.316
Sex (men)		13 (59.1)	223 (63.7)	0.82 (0.34–1.98)	0.663	0.68 (0.27–1.70)	0.406
DM		5 (22.7)	91 (26.0)	0.84 (0.30–2.33)	0.734	0.71 (0.22–2.28)	0.560
HT		11 (50.0)	162 (46.3)	1.16 (0.49–2.47)	0.735	0.92 (0.34–2.49)	0.875
CKD		2 (9.1)	28 (8.0)	1.15 (0.26–5.17)	0.855	0.54 (0.07–4.07)	0.551
Gout		2 (9.1)	9 (2.6)	3.79 (0.77–18.71)	0.102	7.32 (0.85–62.98)	0.070
Dementia		3 (13.6)	10 (2.9)	5.37 (1.36–21.14)	0.016	5.84 (1.34–25.58)	0.019
Arsenism		1 (4.5)	4 (1.1)	4.12 (0.44–38.50)	0.214	3.40 (0.31–36.66)	0.314

DM: diabetes mellitus; HT: hypertension; CKD: chronic kidney disease; COR: crude odds ratio, AOR: adjusted odds ratio; CI: confidence interval. p-value <0.05: two-tailed statistical significance.

### Histopathological profiles of BCC lesions

The histopathologic characteristics of all 403 BCC lesions are shown in Table 4, with 363 (90.1%) lesions being SEBCC and 40 (9.9%) being NSEBCC. NSEBCC had a significantly larger mean dimension measured than that of SEBCC (1.96 vs. 1.10 cm, p = 0.002). SEBCC had a greater depth measured (3.34 vs 3.19 mm), and had a significantly greater Clark level comparing to NSEBCC (p < 0.001). SEBCC had a significantly higher number of high-risk factors for recurrence than did NSEBCC (1.63 vs. 1.04, p < 0.001). Although NSEBCC had a significantly wider surgical margin than that of SEBCC, no significant difference was found regarding the margin free rates.

**Table 4:** Histopathologic characteristics of BCC lesions.

	SEBCC	NSEBCC	p-value
Lesion number (%)	363 (90.1%)	40 (9.9%)	
Dimension (cm), mean ± SD	1.10 ± 1.07	1.96 ± 1.52	p = 0.002*
Depth (mm), mean ± SD	3.34 ± 2.42	3.19 ± 6.54	p = 0.904*
Clark level (%)			p < 0.001
I	0 (0.0)	0 (0.0)	
II	5 (0.4)	3 (11.5)	
III	1 (0.4)	4 (15.4)	
IV	212 (84.1)	17 (65.4)	
V	34 (13.5)	2 (7.7)	
Margin free (%)	258 (91.5)	28 (90.3)	p = 0.826
Closest margin distance (mm), mean ± SD (range)	2.01 ± 1.46 (0.1–9.0)	3.77 ± 2.08 (0.5–8.0)	p < 0.001*
High risk features n. (mean ± SD)	1.63 ± 0.59	1.04 ± 0.77	p < 0.001*
Depth> 2 mm	200 (62.7)	8 (29.6)	p = 0.001
Clark level ≥ 4	247 (97.6)	19 (73.1)	p < 0.001
Lesions on ear or lip (%)	22 (6.1)	0 (0.0)	p = 0.149
Perineural invasion (%)	2 (0.8)	0 (0.0)	p > 0.999
Histopathology (%)			p < 0.001
Nodular	203 (82.2)	16 (55.2)	
Micronodular	6 (2.4)	0 (0.0)	
Superficial	8 (3.2)	7 (24.1)	
Infiltrating or mixed-infiltrating	13 (5.3)	0 (0.0)	
Morpheaform	3 (1.2)	1 (3.4)	
Basosquamous	3 (1.2)	0 (0.0)	
Adenoid	1 (0.4)	0 (0.0)	
Other	10 (4.0)	5 (17.2)	
AJCC Staging (%)			p = 0.314
Stage 1	95 (39.1)	14 (53.8)	
Stage 2	145 (59.7)	12 (46.2)	
Stage 3	3 (1.2)	0 (0.0)	

N: number; SD: standard deviation; AJCC: American Joint Committee on Cancer (AJCC) 7th edition of cutaneous squamous cell carcinoma (cSCC) extrapolated in BCCs. Using two sample t-test (*), otherwise chi-square test or Fisher’s exact test. p-value <0.05: two-tailed statistical significance.

As for histopathologic types, nodular type was the most common type in both groups. Significant differences in the distribution of each subtypes were found between these two groups, with nodular, micronodular, infiltrating or mixed infiltrating, basosquamous, adenoid types being more predominant in SEBCC, while superficial types were more predominant in NSEBCC.

### Risk factors for recurrent BCC

**Figure 1:**
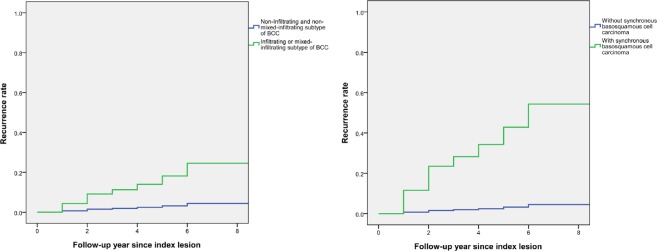
Cox regression survival curve of recurrence rate of BCC associated with synchronous basosquamous cell carcinoma and infiltrating or mixed-infiltrating type BCC.

17 lesions had recurrence during our study period with a total recurrence rate of 4.2% of all patients. All of the recurrent lesions were SEBCC. No case involving distant metastasis was recorded. The associations between patient demographics, comorbidities, synchronous basosquamous cell carcinoma, and recurrence of BCC using Cox proportional hazards model are shown in Table 5. Patients with infiltrating or mixed-infiltrating subtype (aHR = 6.17, CI = 1.07-35.64, p = 0.042) and synchronous basosquamous cell carcinoma (aHR = 16.84, CI = 1.31-216.92, p = 0.030) had a significantly higher association with developing recurrent lesions of BCC. Age, sex, diabetes mellitus, hypertension, and end-stage renal disease had no significant association with developing recurrence after adjustment of covariates in the model. Survival curves regarding recurrence rate of BCC associated with synchronous basosquamous cell carcinoma and infiltrating or mixed-infiltrating type BCC are shown in Fig. 1.

**Table 5:** The adjusted hazard ratio (aHR) of patient demographics and comorbidities associated with recurrence of BCC.

		Recurrence (%)	Non-recurrence (%)	HR (95% CI)	p-value	aHR (95% CI)	p-value
Age of index lesion	<65.0	5 (29.4)	93 (24.1)	1 [Reference]			
	65.0–74.99	4 (23.5)	96 (24.9)	0.77 (0.21–2.89)	0.703	0.43 (0.05–3.37)	0.419
	>= 75.0	8 (47.1)	197 (51.0)	1.30 (0.40–4.22)	0.659	0.39 (0.08–2.06)	0.269
Sex (men)		11 (64.7)	242 (62.7)	0.91 (0.33–2.46)	0.845	0.99 (0.26–3.80)	0.991
DM		5 (29.4)	99 (25.6)	1.38 (0.48–3.93)	0.548	0.45 (0.07–2.77)	0.391
HT		10 (58.8)	178 (46.1)	1.91 (0.72–5.08)	0.194	3.07 (0.70–13.46)	0.137
ESRD		2 (11.8)	12 (3.1)	4.63 (1.04–20.66)	0.045	1.55 (0.10–23.36)	0.751
Infiltrating or mixed-infiltrating subtype		2 (16.7)	11 (4.2)	3.20 (0.62–16.47)	0.165	6.17 (1.07–35.64)	0.042
Synchronous basosquamous cell carcinoma		2 (11.8)	4 (1.0)	12.01 (2.66–54.50)	0.001	16.84 (1.31–216.92)	0.030

DM: diabetes mellitus; HT: hypertension; ESRD: end-stage renal disease; HR: hazard ratio, aHR: adjusted hazard ratio; CI: confidence interval. p-value < 0.05: two-tailed statistical significance. Adjusted HRs based on Cox proportional hazards were calculated after adjustment for age, sex, diabetes, hypertension, end stage renal disease, and synchronous basosquamous cell carcinoma.

## Discussion

In this study, we analyzed the histopathological profiles and outcomes of BCC between sun-exposed and non-sun-exposed areas.

Our study showed that SEBCC had a greater depth and presented with more high-risk features of recurrence than did NSEBCC. The tendency of recurrence in SEBCC may be contributed to a predominant ratio in nodular, morpheaform, basosquamous, micronodular, and infiltrating or mixed infiltrating subtypes. Previous studies reported that anatomic location may favor the development of particular BCC subtypes^15–19^, and certain pathological subtypes were prone to having recurrences^20–22^.

In this study, the risk factors associated with tumor recurrence were synchronous basosquamous cell carcinoma and infiltrating or mixed-infiltrating subtype. Basosquamous cell carcinoma is an uncommon subtype that behaves with a high tendency of local recurrence or metastasis. Changing to basosquamous carcinoma in an initially ordinary BCC has also been reported^23^. Armstrong et al. found that incomplete and close excision margins, infiltrating and micronodular subtypes, and previous excision were strong risk factors for facial BCC recurrence^24^. In our study, no significant differences in the margin free rate between the two groups were found. Current guidelines suggested an excision with 4-mm clinical margins should be made in well-circumscribed, low-risk BCC lesions less than 2 cm in diameter^20,25^. Our study showed a relatively low recurrence rate after excision comparing to previous study^26^, even under a smaller surgical margins in average (2.01 mm in SEBCC, 3.77 mm in NSEBCC).

From our results, we demonstrated that NSEBCC had a significantly larger mean dimension measured and a wider surgical margin than that of SEBCC. This suggests that surgeons always cut a wider surgical margin in NSEBCC due to the large size of the tumors. As nodular, micronodular, infiltrating or mixed infiltrating, basosquamous, and adenoid types were more predominant in SEBCC, superficial types were more predominant in NSEBCC. A total recurrence rate of 4.2% was noted in all patients with no recurrence in patients with NSEBCC. Further subgroup analysis was conducted regarding recurrence rate of the SEBCC and NSEBCC group. SEBCC had a higher recurrence rate than NSEBCC in subgroup which the surgical margin of tumor was less than or equal with 3 mm. No recurrence event was found in subgroup which the surgical margin of tumor was larger than 3 mm. In SEBCC, no significant difference of recurrence rate was found between groups that had surgical margin less than or above 3 mm. This supports our hypothesis that the tendency of recurrence in SEBCC might have to do with its histopathological nature or pathogenic mechanism, rather than its differences in surgical margin width in this study. Altogether, we conclude that unlike SEBCC, NSEBCC might not need a wider surgical margin as current guideline suggested and that a wider safe margin for SEBCC than NSEBCC is indicated.

The existence of NSEBCC may imply a different pathogenesis other than UV light exposure. Our study showed that patients with NSEBCC had a significantly higher association with RA and CKD. Tseng et al. found an increased association with NMSC in RA patients, especially in those using disease-modifying anti-rheumatic drugs^27^. Wang et al. reported that patients with CKD are at a higher risk of developing NMSC^28^, and previous studies implied that accumulation of uremic toxins, oxidative stress, and systemic inflammation in CKD patients may act as important roles in developing malignancy^29,30^. In our study, the different distribution observed in comorbidities between the two groups may suggest a relatively systemic pathogenesis involved in NSEBCC. However, these results should be carefully interpreted given that there was only a limited number of patients in these subgroups and the design of study was a retrospective cohort study rather than a case-control study.

Our study observed an association between immunosuppression status with the development of NSEBCC. Although previous study showed that immunosuppression act as a risk factor of developing non-melanoma skin cancer^31^, we did not observe a significantly higher risk for BCC recurrence among patients with immunosuppression. This discrepancy may result from the following reasons. First, our study design was different from previous studies, as our study aims at comparing patients with SEBCC and NSEBCC, rather than comparing the incidence rate of BCC between immunocompetent and immunosuppressive patients. Since NSEBCC had a lower recurrence rate than SEBCC, this may affect the recurrence rate observed in immunosuppressed patients. Second, the patient’s demographics were different among each study, as our study focusing on Asian population.

Our study showed that patients with dementia had a higher association with developing multiple BCC tumors. A. J. Schmidt et al. reported that NMSC was associated with reductions in risks of dementia^32^. We assumed that the seemingly contrary results may be explained by delayed visits to the hospital or even negligence due to decreased self-awareness on skin condition in patients with cognitive impairment. Selection bias from the surgeons cannot be excluded as well. Further studies to elucidate the causation between dementia and multiple BCC should be done.

This study was conducted at a single medical center with retrospective chart review. To our knowledge, no previous studies have been published, comparing histopathological profiles, risks of multiple tumor and recurrence between SEBCC and NSEBCC in an Asian population. However, this study still has a number of limitations. First, the exact duration and intensity of UV light exposure were impossible to quantify by chart review and the results should be interpreted with caution due to other unmeasured factors attributing to developing multiple BCC or recurrence, such as occupation and lifestyle. Second, lesions that had not yet recurred or recurred after the study period would not have been included in the analysis. Since this was a single medical center study, loss of follow up or referral of patients to local hospitals and clinics may cause potential bias. Third, not all BCC lesions had a comprehensive description of histopathological features in the pathological report. Finally, due to the limited patient numbers and demographic profiles, the results should be carefully applied to other ethnic populations. Future investigation should aim at comparing the optimal surgical margin width in both SEBCC and NSEBCC group under a prospective study design with a larger number of patients and in different ethnic groups.

## Methods

### Data sources

A retrospective review of Kaohsiung Veterans General Hospital’s medical database was performed. Patients with pathologically verified BCC between January 1, 1990 and August 31, 2017 were included (ICD9:173, ICD10: C44.01, C44.11, C44.21, C44.31, C44.41, C44.51, C44.61, C44.71, C44.81, C44.91). All patients in this study were admitted to ward for examinations and surgical excision after receiving biopsy in outpatient department. Patients admitted without pathology to prove BCC with or without recurrence were excluded. Patients were also excluded if they had other skin lesions or tumors, or lost medical records. Both electronic and paper charts within this period of time were reviewed.

### Study design

**Figure 2:**
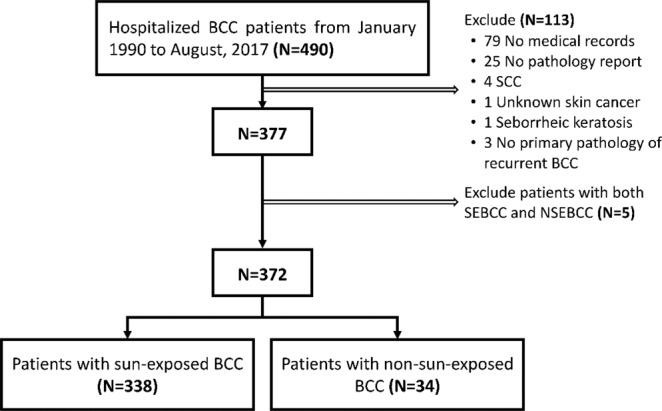
Study design and flowchart of patient selection.

Patients were divided into two groups: those with SEBCC, and those with NSEBCC. Sun-exposed body parts included head and neck, forearms, wrists, and lower legs. In order to compare the differences between the two groups, patients with both SEBCC and NSEBCC were excluded from this study. Figure 2 shows the patient selection flowchart.

Data were derived from chart review and pathology reports, including sex, age at diagnosis, relevant medical comorbidities, anatomic tumor site, surgical data, staging, and histopathological profile. Multiple BCC, recurrence, and metastasis were also recorded. Multiple BCC is defined as more than one tumor diagnosed at different anatomic sites synchronously or asynchronously. Recurring lesions were observed from the time period of January 1990 until the patient’s latest visit to the hospital and are defined as a tumor re-occurring in the primary tumor site after excision.

### Histopathological profile

Both the width and depth of the lesions were documented according to the pathology report. Pathological type, Clark level, tumor staging, surgical margin status, and high-risk features of recurrence were also obtained from the pathology report. The staging of BCC is defined according to the American Joint Committee on Cancer (AJCC) 7^th^ edition of cutaneous squamous cell carcinoma (cSCC) extrapolated in BCCs.

### Statistical analysis

Differences in the distribution of demographic data, comorbidities and BCC characteristics were compared using the t-test for continuous variables, and the Pearson χ^2^ test or Fisher’s exact test for categorical variables. Logistic regression models were used to calculate crude and adjusted odds ratios (aOR) with 95% confidence intervals (CI) for the development of multiple BCC lesions. No significant interactions were found among variables that were selected for the multivariate logistic regression model. Cox proportional hazards model was used to estimate hazards ratios (HR) and 95% CIs for the recurrence of BCC lesions. SPSS statistical software, version 19.0 for Windows, was used for all data analysis. All methods were performed in accordance with the relevant guidelines and regulations. This retrospective study adhered to the tenets of the Declaration of Helsinki and was approved by the Institutional Review Board of Kaohsiung Veterans General Hospital (approved number: VGHKS19-CT7-02). A waiver of informed consent was granted by the approving Institutional Review Board.

## Conclusion

In conclusion, our study found that SEBCC had a higher recurrence rate with a significantly different tumor characteristic from NSEBCC and a greater tumor depth than NSEBCC. As the superficial type is predominant in NSEBCC and no recurrence in NSEBCC patients was found in this study, we suggested NSEBCC might not need a wider surgical margin as current guideline suggested and a wider safe margin for SEBCC than NSEBCC is indicated. The risk factors for BCC recurrence included infiltrating or mixed-infiltrating subtype and the existence of synchronous basosquamous cell carcinoma. Patients with NSEBCC had a significantly higher association with chronic kidney disease and rheumatoid arthritis than with those in the SEBCC group.

## Data Availability

The datasets generated during and/or analyzed during the current study are available from the corresponding authors on reasonable request.
